# Synthesis of Amorphous Carbon Film in Ethanol Inverse Diffusion Flames

**DOI:** 10.3390/nano8090656

**Published:** 2018-08-24

**Authors:** Jie Zhu, Fang Li, Guannan Liu, Dong Liu, Qiongyu Li, Erjun Kan

**Affiliations:** 1 MIIT Key Laboratory of Thermal Control of Electronic Equipment, School of Energy and Power Engineering, Nanjing University of Science and Technology, Nanjing 210094, China; jiezhu@njust.edu.cn (J.Z.); liuguannan0925@126.com (G.L.); 2 Advanced Combustion Laboratory, School of Energy and Power Engineering, Nanjing University of Science and Technology, Nanjing 210094, China; 3 Department of Applied Physics and Institution of Energy and Microstructure, Nanjing University of Science and Technology, Nanjing 210094, China; lifang@njust.edu.cn (F.L.); ekan@njust.edu.cn (E.K.)

**Keywords:** flame synthesis, amorphous carbon film, inverse diffusion flame

## Abstract

Recently, carbon nanomaterials have attracted significant attention due to their remarkable physical and chemical properties. The preparation methods and applications of the carbon nanomaterials have developed rapidly. In this study, the flame synthesis of amorphous carbon film grown on copper foil in an ethanol inverse diffusion flame was presented. The effects of ethanol flow rate, the copper foil location in flame and growth time were investigated in detail. The growth status of the synthetic amorphous carbon film was analyzed by an optical microscope and HRTEM (high resolution transmission electron microscope). Raman spectroscopy and XRD (X-ray diffraction) were used to characterize the structure of the carbon film. The roughness of the carbon film was determined by AFM (atomic force microscopy). As the ethanol flow rate increased and the copper foil moved upwards in the flame, the area of the synthetic amorphous carbon film increased. The roughness of carbon films with the growth time of 30 s and 2 min were smaller. In addition, the synthetic amorphous carbon film exhibited a certain degree of flexibility and visual transparency. Through the study, a reference could be provided to find the optimum condition for the flame synthesis of satisfactory amorphous carbon film. For these experiments, when the ethanol flow rate reached 2 mL/min, the copper foil was located on the top of the flame and the growth time was 2 min, an amorphous carbon film with higher quality could be obtained.

## 1. Introduction

Recently, materials in nanoscale have attracted more and more attention because of their unique physical and chemical properties, and the preparation and application of nanomaterials have become research hotspots for scientists. Carbon is usually abundant and easily available on the earth, so the studies on carbon nanomaterials have also emerged and developed rapidly. In the past few years, the cutting-edge hot topics in various subjects involved carbon nanomaterials such as carbon nanotubes (CNTs) [1,2,3,4] and graphene [5,6,7,8,9,10], especially many of the extraordinary mechanical, electrical and optical characteristics they possessed [11,12,13,14,15].

Among these nanostructures mentioned above, there were thin-films called amorphous carbon (A–C) films, whose structure and properties were both between those of graphite and diamond. They were amorphous and microcrystal carbon films composed of C with sp^2^ and sp^3^ hybridization [16]. By adjusting the ratio of the content of sp^2^ and sp^3^ bonds, carbon films could be obtained with different properties [17]. For example, amorphous carbon films having a significant fraction of C–C sp^3^ bonds generally exhibited high hardness, large optical gap and high resistivity [18,19]. These carbon structures were defined as diamond-like carbon (DLC) films. Conversely, amorphous carbon films which had a greater content of C=C sp^2^ bonds were referred to as graphite-like carbon (GLC) films. They usually revealed good wear performance, conductivity and small optical gap [20,21]. Since Aisenuberg et al. published a paper on amorphous carbon films in the United States in 1971 [22], amorphous carbon films have started to be applied to wear-resistant coatings, optical windows, integrated circuits and field emission devices, even biomedical materials [18,23,24,25].

At present, the main methods for preparing carbon nanomaterials include arc discharge, pulsed laser deposition and chemical vapor decomposition (CVD) [6,26,27,28,29]. Although the methods above and their technologies have developed relatively well, there are still some problems that limit large-scale applications. Providing a vacuum or low-pressure environment and ensuring a lengthy synthesis time imposed high requirements on the synthesis technology and its equipment, and the cost required for synthesis was also increased. Some scholars also proposed a novel and potential method, called as flame synthesis. It utilized different carbon-based fuels to achieve continuous, large-area and low-cost synthesis [2,30]. The flame could provide both the heat source and the carbon source [5]. During the reactions, the flame produced hydrocarbons, carbon monoxide and other precursors needed for synthesis. Moreover, high temperature of the flame could also be used to heat the substrate without any additional heat source [31,32]. Premixed, partially premixed and non-premixed flames have all been tried in the synthesis of carbon nanomaterials [2], and many studies have shown the high efficiency of flame synthesis [33,34,35].

In this study, the flame synthesis of amorphous carbon film on copper foil with a short time was reported, which utilized pre-evaporation gas inverse diffusion (non-premixed) ethanol flame in open-atmosphere environments. Ethanol was easily available and economical as a common fuel, and the open environments ensured the low cost of overall experiment as well. The inverse diffusion flame burner used here was different from the normal diffusion flame burner, and the oxidant was in the center of the inverse diffusion flame burner with fuel surrounding it. The inverse diffusion flame contained a large amount of carbonaceous species, and a very low molar fraction of oxygen which formed a reducing atmosphere [31,32,36]. In other words, carbon could be separated from oxygen, and the formation of soot in this flame was also less than in the normal diffusion flame [37], which was more favorable to the synthesis of carbon nanomaterials. There were few literatures on the synthesis of amorphous carbon films using the normal diffusion flame burner, by far. Furthermore, the synthesis configuration utilizing a bigger burner or a multiple inverse diffusion flame burner was expected to enable the scalable production of amorphous carbon film [38].

This study aimed to present a method for the synthesis of amorphous carbon film which was simple, economical and efficient. The different synthesis conditions were compared and optimized by adjusting experimental parameters such as growth time, ethanol flow rate and the copper foil location in flame. However, there were many factors affecting the synthesis of amorphous carbon film. Therefore, these factors needed to be examined before the application of the carbon film for practical use. In addition, the measurement and analysis of the properties of the synthetic carbon film, that is, the application aspect is proposed as one of our future studies.

## 2. Experimental Section

### 2.1. Experimental System

**Figure 1:**
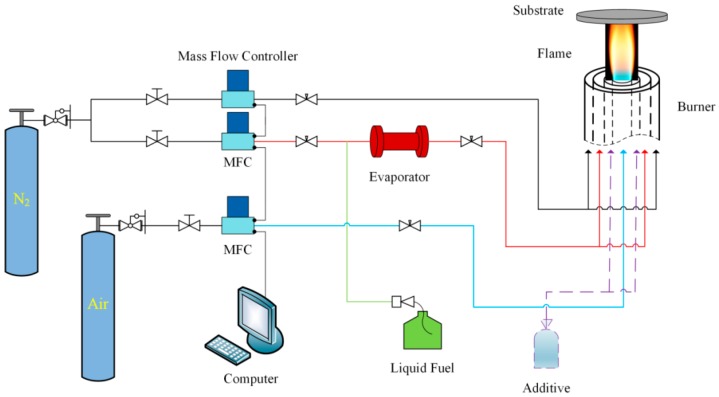
Schematic diagram of experimental system.

The entire experimental system consisted of a circular coaxial inverse diffusion flame burner, an evaporator, three mass flow controllers, and a liquid injection pump, as shown in Figure 1. Air was delivered from the center of the inverse diffusion flame burner, and fuel (ethanol) was provided from the middle annular channel. Before entering the burner, ethanol needed to be vaporized by the evaporator and carried by a small amount of nitrogen (carrier nitrogen). Protection nitrogen was transported from the outermost channel. The large flow rate of protection nitrogen not only prevented the osmosis of oxidant from outside environment, but also stabilized the flame while directed the optimum gas phase condition (temperature and species) to the substrate (copper foil) for synthesis [36]. The whole fuel flow path was equipped with temperature control devices to ensure that the evaporated ethanol would not be cooled and liquefied before reaching the burner nozzle. 

The copper foil (2 cm × 2 cm, Alfa Aesar, Thermo Fisher Scientific Inc., Heysham, Lancashire, UK, 0.025 mm, 99.8%) was placed above the burner nozzle, and the position was controlled by a three-dimensional displacement platform. Before the synthesis experiments, electrolytic polishing was needed to remove oxide layers on the copper foil surface and expose internal active metal for catalyzing the growth of carbon film.

After the reactions, the air channel was turned off and the flame was extinguished simultaneously, but the flow rates of ethanol and nitrogen were still maintained during cooling.

### 2.2. Experimental Conditions

Table 1 shows specific conditions of the synthesis experiment. The height measured the vertical distance between the copper foil and the burner nozzle. With the increase of ethanol flow rate, the flow rate of carrier nitrogen increased in proportion. It was to make sure that the evaporated ethanol could be smoothly carried to the burner nozzle for combustion and synthesis. In addition, the temperature control devices and evaporator were set to 120 °C.

**Figure 2:**
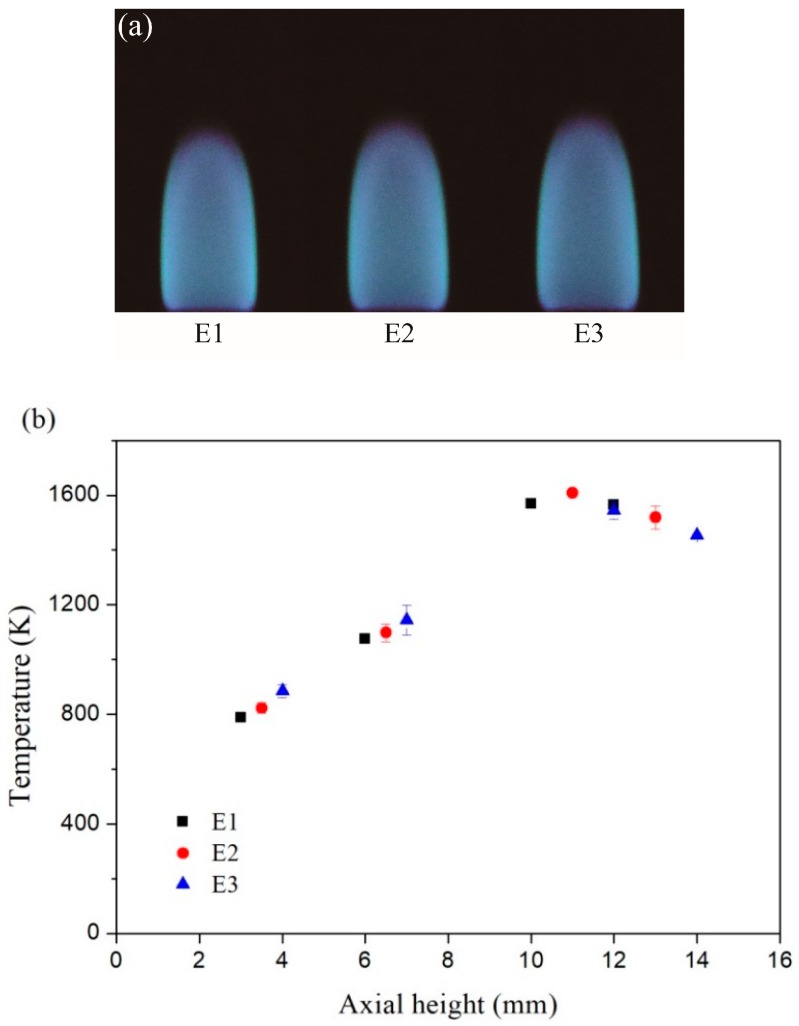
(**a**) Inverse diffusion flame images; (**b**) Corresponding measured temperatures with error bars at different axial height.

The images of the inverse diffusion flame and the corresponding axial temperatures at the flame center are exhibited in Figure 2a,b, respectively. The temperatures were measured by an uncoated B-type thermocouple (Omega Engineering, Inc., Stamford, CT, USA) and the values were corrected for radiation losses from the surface of the thermocouple. The total heights of the inverse diffusion flames under three different conditions were different. For each condition, the four different height values listed in Table 1 corresponded to the lower, middle, upper and the top of the flame severally. As seen in Figure 2b, the temperature at the upper of the flame was the highest, and the temperature at the top of the flame was lower slightly. The temperature at the middle and lower of the flame were all below 1200 K.

### 2.3. Characterizations and Transfer

The growth status of the carbon films on the copper foil surfaces were analyzed by an optical microscope (Metallurgical Microscope, MV5000, Nanjing Jiangnan Novel Optics Co., Ltd., Nanjing, China) [38]. A high resolution transmission electron microscope (HRTEM, Tecnai G2 F30 S-TWIN, FEI, Hillsboro, OR, USA) was used to analyze the microstructure of the synthetic carbon films. The grid used for HRTEM analysis was lacey formvar carbon film coated grid with 200 meshes, which allowed the sample to be viewed without a background. The TEM/HRTEM magnification calibration was required before the formal inspection of the synthetic samples. A standard sample (a copper mesh with a known mesh size) was used as a scale during calibration. The structure and bonding properties of the carbon film were characterized by Raman spectroscopy (Renishaw inVia, Renishaw plc., Gloucestershire, UK, laser excitation 532 nm). The integration time for the Raman spectra was 20 s, and the laser power was 2 MW. In addition, the groove density of grating used was 1800 g/mm. The crystal structure of the carbon film was analyzed using X-ray diffraction (XRD, D8 Advance, Bruker-AXS, Karlsruhe, Germany) with CuK_α_ radiation over a 2θ range of 10°–80°. Atomic force microscopy (AFM, Diension Icon, Bruker, Karlsruhe, Germany) was used to measure the surface morphology and roughness of the carbon film. The type of AFM probes was ScanAsyst-Fluid (Bruker, Karlsruhe, Germany). The probes nominal spring constant was 0.7 N/m, and tip radius was 2 nm. The imaging mode was scanasyst-imaging mode. A standard sample (surface topography references, VGRP-15M, Bruker, Karlsruhe, Germany, 180 nm step-height, 10 μm pitch) was scanned to confirm that the image was error free before the synthetic samples were formally tested. The film thickness of the carbon film was characterized by scanning electron microscope (SEM, S-4800, Hitachi Limited, Tokyo, Japan) and the elemental composition of the carbon film was analyzed using X-ray photoelectron spectroscopy (XPS, PHI 5000 VersaProbe, ULVAC-PHI, Inc., Chigasaki, Kanagawa, Japan).

The carbon film needed to be transferred from the copper foil onto a Si/SiO_2_ substrate for Raman or XRD analysis, and onto a TEM grid for TEM/HRTEM study. Firstly, poly-methyl methacrylate (PMMA) was spin-coated on the front side of the copper foil (the side that touched the flame in experiment). Then, the back side was placed on a prepared ammonium persulfate solution to remove copper and washed with deionized water three times. After copper was etched, the carbon film was carefully placed on a Si/SiO_2_ substrate or fished out by a TEM grid, and dried under vacuum. Finally, the PMMA attached to the carbon film surface was removed in acetone, and then ethanol and deionized water were used to rinse the sample [39]. Raman measurements of transferred carbon film on Si/SiO_2_ substrate provided much better information neglecting the Cu substrate effect.

## 3. Results and Discussion

### 3.1. Growth of Carbon Films on Copper Foils

**Figure 3:**
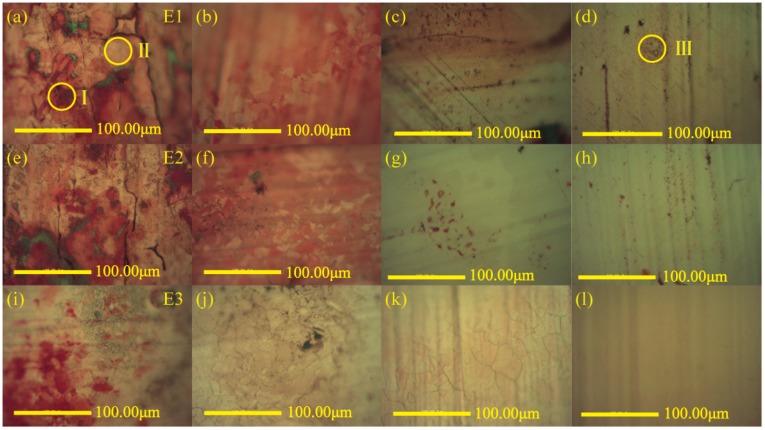
Optical microscope images of carbon films on copper foils at different ethanol flow rates and different heights with the growth time of 5 min: (**a**) 1.0 mL/min, 3.0 mm (E1-1); (**b**) 1.0 mL/min, 6.0 mm (E1-2); (**c**) 1.0 mL/min, 10.0 mm (E1-3); (**d**) 1.0 mL/min, 12.0 mm (E1-4); (**e**) 1.5 mL/min, 3.5 mm (E2-1); (**f**) 1.5 mL/min, 6.5 mm (E2-2); (**g**) 1.5 mL/min, 11.0 mm (E2-3); (**h**) 1.5 mL/min, 13.0 mm (E2-4); (**i**) 2.0 mL/min, 4.0 mm (E3-1); (**j**) 2.0 mL/min, 7.0 mm (E3-2); (**k**) 2.0 mL/min, 12.0 mm (E3-3); (**l**) 2.0 mL/min, 14.0 mm (E3-4).

Figure 3 revealed the optical microscope images of copper foils surfaces after synthesis at different fuel (ethanol) flow rates and different heights of copper foils (growth time was 5 min). Before using the optical microscope for observation, the resulting sample was heated at 150 °C in open-atmosphere environments for 3 min firstly. Selective oxidation of copper foil surface was convenient for the observation. Low-temperature oxidation could not change the characteristics of carbon film. Instead, copper foil was turned into copper oxide, making the carbon film easier to find due to color contrast [40]. The areas not covered by films appeared red, while the areas covered by films showed yellow, just like the zones marked with circle І and ІІ, respectively, in Figure 3a.

As a whole, at the three different ethanol flow rates, the color of the red areas became lighter and the red areas were also reduced when the copper foils were moved upwards in the flames, which indicated that carbon films formed gradually. The lower of the inverse diffusion flame was closer to the burner nozzle where air (oxidant) flowed past. The copper foil was easily oxidized as a result of the higher oxidant content [36]. Conversely, at the upper of the flame, especially at the top, there were abundant reduction species such as CO and H_2_. They were accumulated to form a reducing atmosphere [41], which was favorable for the synthesis of carbon films.

Figure 3a–d displayed many red areas on copper foil surfaces at the four different heights of copper foils, when the ethanol flow rate was a minimum value of 1 mL/min. It indicated that there were few films formed on copper foils. Besides, there were also some obvious black or gray areas in the field of vision just like the zone marked with circle ІІІ in Figure 3d. This was presumably because some other carbon structures such as soot emerged and adhered to the copper foil rather than the carbon film as we expected [8]. As shown in Figure 3e–h, when the flow rate of ethanol increased to 1.5 mL/min, the red areas on copper foils surfaces were reduced to varying degrees at the four corresponding copper foils locations, especially at the upper and the top of the flame. Compared with the conditions above, the flow rate ratio of air and fuel decreased, and accordingly, the content of oxidant inner the flame was also reduced, which was more conducive to the synthesis of carbon films [36]. When the ethanol flow rate was a maximum value of 2 mL/min, even if the copper foil were inserted into the middle and lower of the flame, the red areas on the copper foil surfaces were small, as seen in Figure 3i,j, but the black or gray areas still existed, which pointed out that there were some impurities on the carbon film.

From the descriptions above, the following results could be obtained. The synthetic carbon film was better when the ethanol flow rate reached 2 mL/min and the copper foil was located on the top of the flame. Under this condition, the optical microscope image of the copper foil surface exhibited a uniform yellow zone and no visible black or gray impurities in Figure 3l.

**Figure 4:**
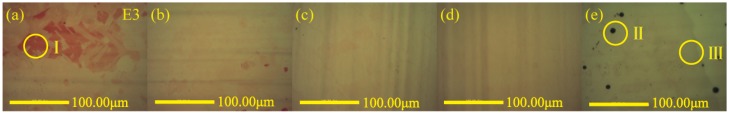
Optical microscope images of carbon films on copper foils with different growth time at ethanol flow rate of 2 mL/min and height of 14.0 mm: (**a**) 10 s (E3-5); (**b**) 30 s (E3-6); (**c**) 2 min (E3-7); (**d**) 5 min (E3-4); (**e**) 10 min (E3-8).

Figure 4 showed the optical microscope images of carbon films on copper foils for different growth time. The ethanol flow rate was maintained at 2 mL/min, and the copper foil was inserted into the top of the flame. Figure 4a corresponded to the growth time of 10 s, and most zones appeared yellow. It indicated that the growth of the carbon film occurred in such a short time. However, some areas still appeared light red just like the zone marked with circle І in Figure 4a. Either there was no carbon film, or the formation of the carbon film was still in its initial stage. In other word, the carbon film was thin and had not yet formed a complete continuous sheet [40]. With the increase of the growth time, yellow areas in Figure 4b–d connected intact gradually without any obvious red area or gray boundary.

Nevertheless, the growth of carbon film showed a self-limited characteristic similar to the graphene growth on copper foil [6,41]. Scholars found that the growth of graphene on copper was a surface-catalyzed process rather than a precipitation process like on nickel [6]. Only a small amount of carbon was absorbed into copper foil, in consequence the growth of graphene on copper foil was not caused by the out-diffusion of carbon atoms [31]. Analogously, the formation of carbon film was attributed to a surface adsorption mechanism of carbon on copper foil [41]. Once the surface of copper foil was covered by carbon film, the growth would end or the film would grow at a very low rate.

When the growth time increased to 10 min, there were some black spots and gray areas on the copper foil surface, just like the zones marked with circle ІІ and ІІІ in Figure 4e. It indicated the formation of other additional carbon structures attaching to the carbon film surface, like soot. After the longer synthesis time of 10 min, the carbon film had covered the whole copper foil surface, and more carbon could not come to contact with copper. As a result, the growth of the carbon film stopped basically [41], replaced by the formation of soot and other carbon structures [8].

In short, the growth time of 30 s, 2 min and 5 min were more suitable for the synthesis of carbon films. The growth time of 30 s, 2 min and 5 min were longer than 10 s, so the carbon film could be formed and connected into a complete continuous sheet relatively. But there was no obvious impurity deposited on the carbon film surface due to the relatively shorter growth time. However, the observation only by optical microscope was not enough to compare the growth of the carbon films, so more characterization and further analysis were needed and showed in the following.

### 3.2. Microstructure Analysis of Carbon Films

**Figure 5:**
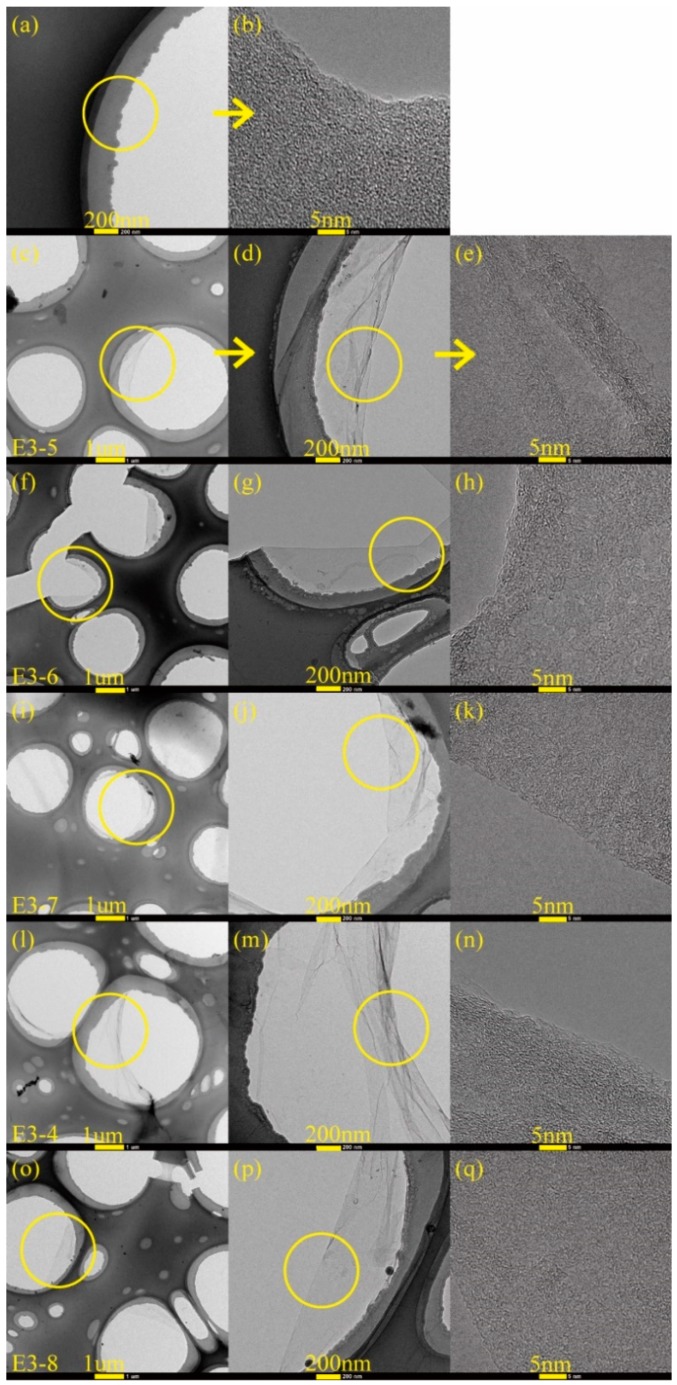
TEM images of carbon films with different growth time at ethanol flow rate of 2 mL/min and height of 14.0 mm: (**a**) TEM grid; (**c**,**d**) 10 s (E3-5); (**f**,**g**) 30 s (E3-6); (**i**,**j**) 2 min (E3-7); (**l**,**m**) 5 min (E3-4); (**o**,**p**) 10 min (E3-8). HRTEM images of carbon films with different growth time at ethanol flow rate of 2 mL/min and height of 14.0 mm: (**b**) TEM grid; (**e**) 10 s; (**h**) 30 s; (**k**) 2 min; (**n**) 5 min; (**q**) 10 min.

**Figure 6:**
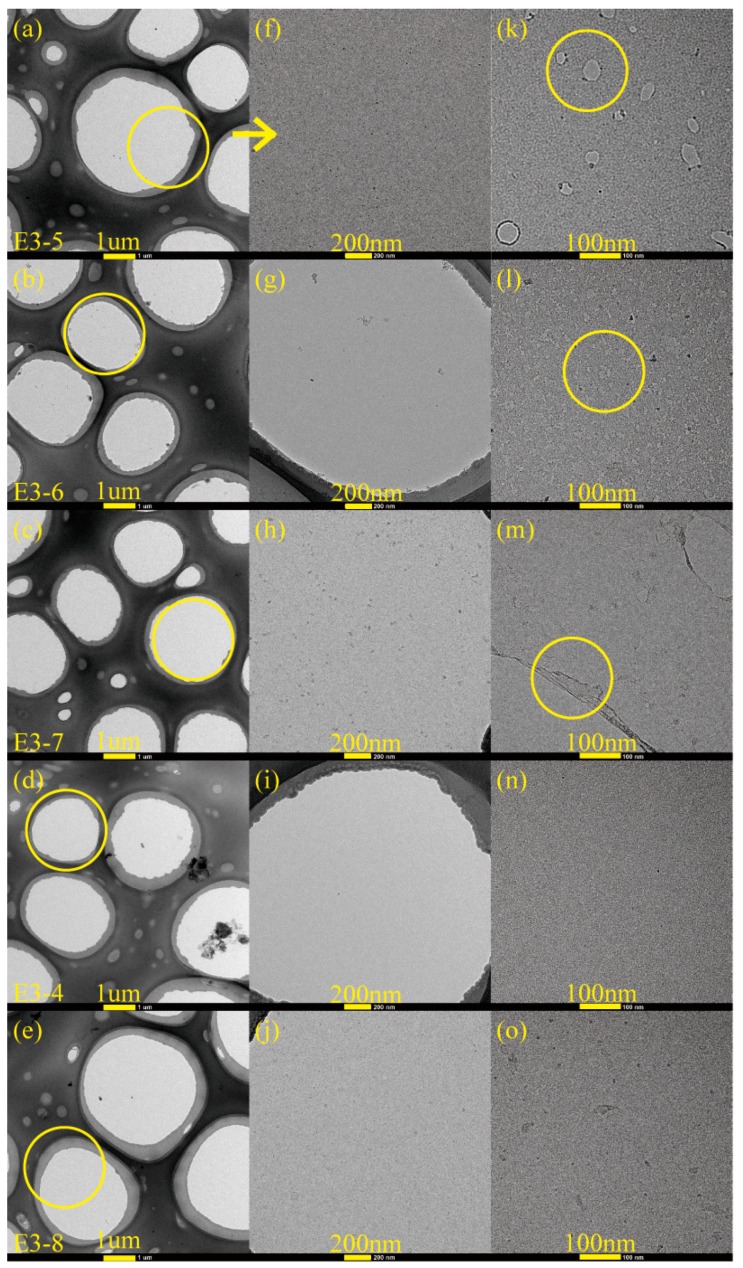
TEM images of carbon films with different growth time at ethanol flow rate of 2 mL/min and height of 14.0 mm: (**a**,**f**) 10 s (E3-5); (**b**,**g**) 30 s (E3-6); (**c**,**h**) 2 min (E3-7); (**d**,**i**) 5 min (E3-4); (**e**,**j**) 10 min (E3-8). HRTEM images of carbon films with different growth time at ethanol flow rate of 2 mL/min and height of 14.0 mm: (**k**) 10 s; (**l**) 30 s; (**m**) 2 min; (**n**) 5 min; (**o**) 10 min.

Figure 5 and Figure 6 exhibited the TEM and HRTEM images of carbon films formed on copper foils for different growth time (ethanol flow rate was 2 mL/min, the copper foil located on the top of the flame). Among them, Figure 5a,b were the images of TEM grid (without sample). As seen from the two figures, each mesh of TEM grid had a supporting carbon layer carried by itself, and the center of each mesh was a blank zone without any substance attachment. Therefore, the following carbon films found in the middle of meshes were obtained from synthesis experiments.

The formation of carbon films occurred for the five different growth time. Figure 5c–q revealed the edges of the synthetic carbon films. Amorphous and disordered structures of the carbon films were observed by Figure 5e,h,k,n,q. For Figure 5d,g,j,m,p, there were some overlaps and wrinkles, and the back side layer could be clearly identified through the previous layer in overlapping area. In addition, although some zones of the carbon film covered TEM grid, the skeleton of TEM grid could still be seen through the carbon film. These further explained the ultra-thin and visual transparency of the carbon film.

Figure 6a–j,n,o exhibited the middle zones of carbon films. These carbon films were complete and continuous in the main, especially for the growth time of 10 min and 5 min. It was worth noting that the carbon films here undergone transfer and washing processes before, which proved their robustness and flexibility. Nevertheless, for the growth time of 2 min, 30 s and 10 s, rare but specific places appeared on the carbon films just like the zones marked with yellow circles in Figure 6k–m. For Figure 6m, there were two small cracks. The probable reason was that some parts of the carbon film were thinner relatively and were easy to be torn during the transfer and washing processes. But this was only a very rare phenomenon, the synthetic carbon film with the growth time of 2 min was almost complete. As shown in Figure 6k, the carbon film was not very complete, which indicated that the 10 s growth time was too short to merge together for individual zones of the carbon film after local growth [40]. A similar case could also be seen in Figure 6l. The difference was that the gaps showed smaller as a result of the longer growth time of 30 s.

### 3.3. Raman Spectra and XRD Analysis

Raman spectroscopy is one of the important and non-destructive techniques used to analyze the microstructure of amorphous carbon film [16]. The Raman peak of single crystal graphite was located at 1580 cm^−1^, which was called G peak. With the increase of disorder degree in carbon film, D peak appeared around 1350 cm^−1^. The two peaks were distinct characteristic ones that usually revealed on Raman spectrum of amorphous carbon film [16].

**Figure 7:**
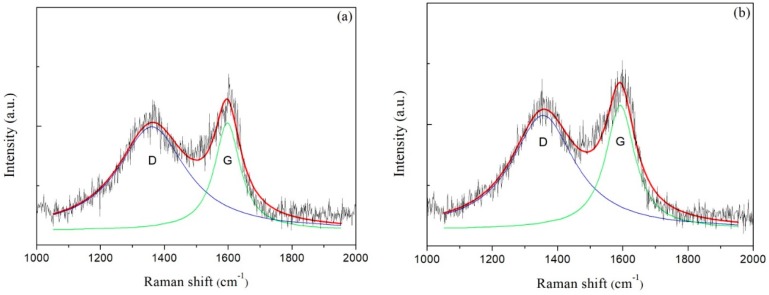

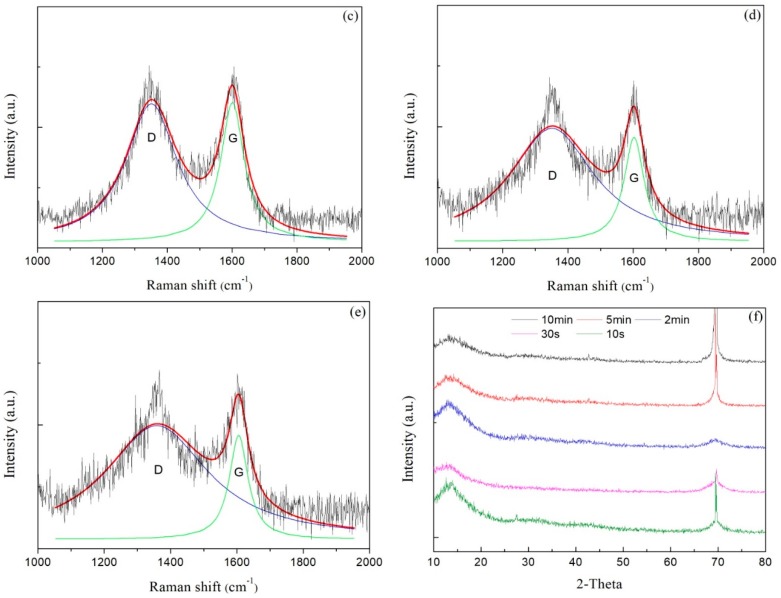
Raman spectra of carbon films with different growth time at ethanol flow rate of 2 mL/min and height of 14.0 mm: (**a**) 10 s (E3-5); (**b**) 30 s (E3-6); (**c**) 2 min (E3-7); (**d**) 5 min (E3-4); (**e**) 10 min (E3-8); (**f**) XRD patterns of carbon films with different growth time at ethanol flow rate of 2 mL/min and height of 14.0 mm.

Raman spectra of carbon films with different growth times (ethanol flow rate was 2 mL/min, the copper foil located on the top of the flame) are shown in Figure 7a–e. The corresponding data of G peak and D peak positions, the ratio of the peak areas of D and G peak (*I*_D_/*I*_G_) and the full width at half maximum of G peak (FWHM_G_) are listed in Table 2. Raman spectra were collected at three different locations for each sample, and the relevant data of each sample were averaged from the Raman spectra data at these three different locations. These Raman spectra shown in Figure 7 were one of the Raman spectra of each sample. The composition and structure of the carbon film were analyzed by the details of the peaks after fitting the spectra through Lorentz peak mode. It was generally recognized that *I*_D_/*I*_G_ corresponded to the ratio of sp^2^/sp^3^ in carbon film [42]. 

As seen in Table 2, G peaks were located around 1590–1610 cm^−1^, and D peaks were located around 1350–1360 cm^−1^. In theory, these films were amorphous carbon films [17]. And G peaks FWHM were lager, which represented high levels of disorder in the carbon films [43]. When the growth time increased from 10 s to 30 s, G peak and D peak positions moved in low wavenumber direction, and I_D_/I_G_ decreased, and FWHM_G_ increased. These changes indicated that the content of sp^3^ bonds in the carbon film increased while the content of sp^2^ bonds reduced, which resulted in the increase of disorder degree. The carbon film changed from graphite-like to diamond-like concurrently. It was not hard to speculate that the structure of the carbon film was no longer loose. There were more edges, wrinkles and multiple defects that emerged which were suitable for additives adhering to improve some properties of films [36]. When the growth times were 30 s and 2 min, *I*_D_/*I*_G_ of carbon films were consistent with the reported values by other methods, such as plasma-enhanced chemical vapor deposition [30] and direct current magnetron sputtering [19], but these methods had a certain level of demand for energy supply and low-pressure environments. Huang et al. used high-power impulse magnetron sputtering (HiPIMS) coupled with a direct-current magnetron sputtering (dcMS) in parallel to synthesize amorphous carbon films [20]. The *I*_D_/*I*_G_ values of the synthetic carbon films were 3–4, which showed that the carbon films were more inclined to graphite-like carbon films.

With a continuous increase in growth time, the carbon film gradually covered the entire surface of copper foil. As mentioned in the analysis of optical microscope images above, the growth of the carbon film stopped or the carbon film grew very slowly. Moreover, the experimental conditions were the same besides growth time; hence, the structure and chemical bonding state of carbon films with longer growth time might not make a big difference. However, from the data in Table 2, G peak and D peak positions moved in high wavenumber direction, and *I*_D_/*I*_G_ increased, as the growth time varied from 2 min to 10 min. The reasons might be as follows. First, although the growth of the carbon film was almost stopped after the growth time reached 2 min from the previous analysis and Figure 6, the degree of order, graphitization and the sp^2^ bonds content still increased due to the high temperature in the flames for a longer time. The second reason was that other additional carbon structures such as soot were formed on the carbon film surface. The increase of deposition time brought about the high level of order in soot, and the degree of graphitization and the sp^2^ bonds content increased likewise [44].

Figure 7f exhibited XRD patterns of carbon films with different growth time (ethanol flow rate was 2 mL/min, the copper foil inserted into the top of the flame). In the entire diffraction angle (2θ) range of 10°–80°, the peak before 2θ = 15° was the characteristic peak of the substrate used for XRD. There was a broad and small peak around 30°, but not a characteristic peak of crystal. In addition, the peak at about 68° was corresponding to the diffraction peak of Si/SiO_2_ substrate. These descriptions showed that the resulting carbon films were amorphous carbon films.

### 3.4. AFM Images Analysiss, Film Thickness and Elemental Composition

**Figure 8:**
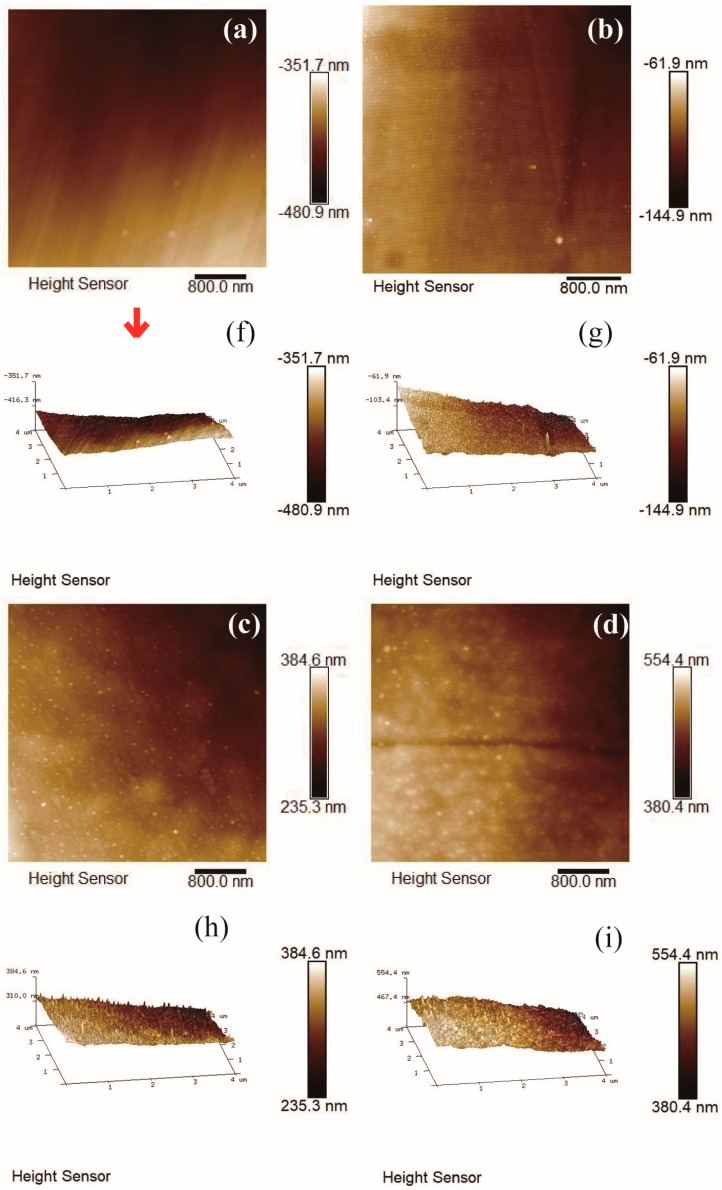

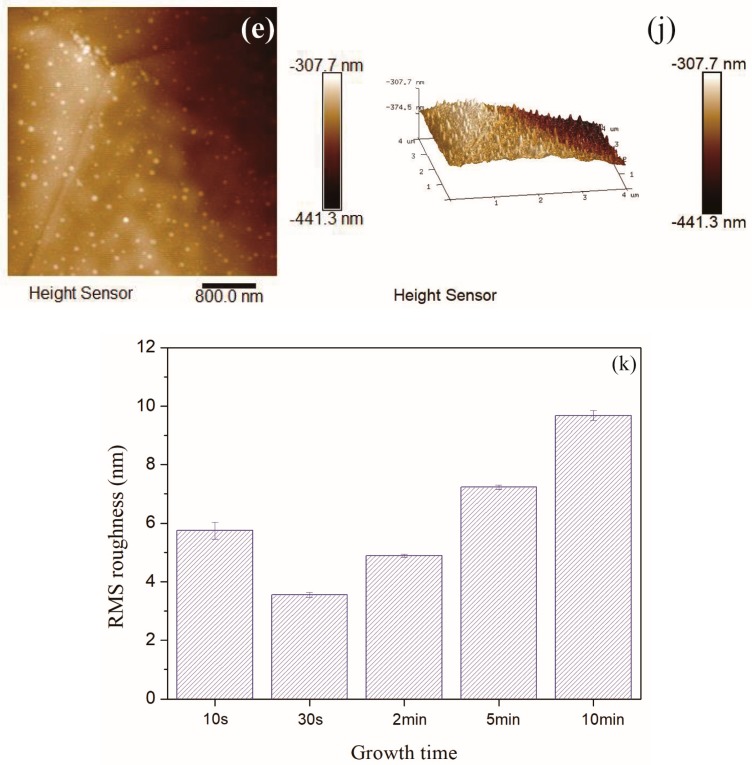
4 μm × 4 μm AFM 2D images of carbon films with different growth time at ethanol flow rate of 2 mL/min and height of 14.0 mm: (**a**) 10 s (E3-5); (**b**) 30 s (E3-6); (**c**) 2 min (E3-7); (**d**) 5 min (E3-4); (**e**) 10 min (E3-8). 4 μm × 4 μm AFM 3D images of carbon films with different growth time at ethanol flow rate of 2 mL/min and height of 14.0 mm: (**f**) 10 s (E3-5); (**g**) 30 s (E3-6); (**h**) 2 min (E3-7); (**i**) 5 min (E3-4); (**j**) 10 min (E3-8); (**k**) Corresponding RMS roughness.

The typical morphology of amorphous carbon film contained island-like structures in the order of nm scale on the surface of carbon film. Figure 8a–j depicted the 4 μm × 4 μm AFM images of carbon films with different growth time (ethanol flow rate was 2 mL/min, the copper foil inserted into the top of the flame), and the corresponding root mean square (RMS) roughness was summarized in Figure 8k. Each AFM image was processed using NanoScope Analysis software to obtain RMS roughness value of the 256 × 256 points in the 4 μm × 4 μm area. Here, RMS roughness was the root mean square average of the height deviations taken from the mean data plane [45,46].

It could be seen that RMS roughness of the carbon film increased with the increase of the growth time, except the case of 10 s growth time. The growth time of 10 s was short, and some areas of the carbon film could not be connected completely in time. Therefore, the surface of entire sample was not smooth and the RMS roughness was slightly larger. RMS roughness of carbon films with growth time of 30 s and 2 min were smaller relatively. The values were similar to the reported values by other technologies, such as direct current magnetron sputtering [19] and hot-filament chemical vapor deposition [17]. The difference was that the heat source was provided here directly by the flame for synthesis under normal pressure in this experiment. When the growth time was more than 2 min, the carbon film surface became loose, and there were obvious island-like cluster structures appeared on the surface. It indicated the decrease of sp^3^ bond in the films structures [17]. This was consistent with the results of previous Raman analysis. As the growth time increased, the content of sp^2^ bonds increased, the carbon film tended to be graphitized, and the surface of the carbon film became uneven. From Figure 8c–e, some granular substances (soot and other impurities) could be found on the carbon films surfaces. This was one of the reasons why RMS roughness of the carbon film was larger.

**Figure 9:**
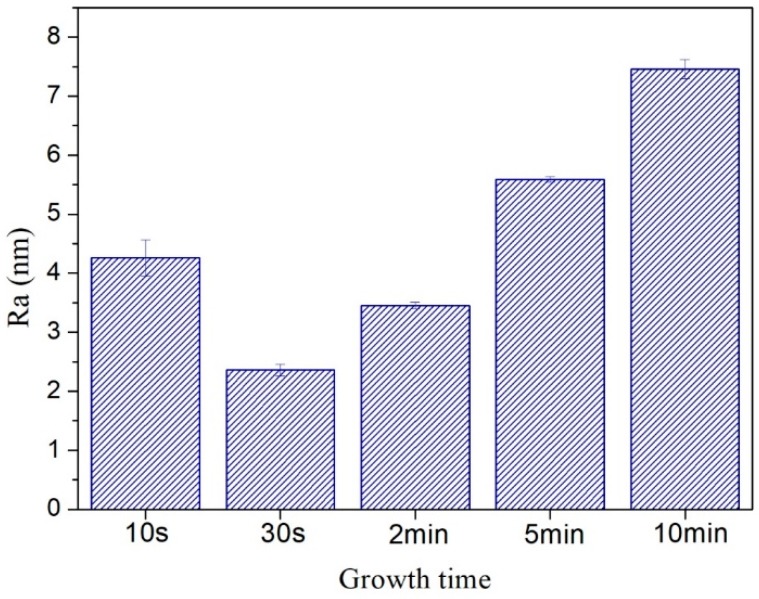
Average roughness of carbon films with different growth time (10 s/30 s/2 min/5 min/10 min) at ethanol flow rate of 2 mL/min and height of 14.0 mm.

In order to more comprehensively analyze the roughness of the carbon film surface, the average roughness (Ra) values were summarized in Figure 9. Here, Ra was the arithmetic average of the absolute values of the surface height deviations measured from the mean plane [45,46]. The average roughness (Ra) values were slightly smaller than the RMS roughness values, but the basic law of roughness values variation with growth time was consistent. Moreover, there was no flattening treatment in Figure 8 to prevent image distortion. The RMS roughness values after “Flatten” treatment were slightly smaller than the values in Figure 8k, but the basic law of RMS roughness values variation with growth time did not change.

**Figure 10:**
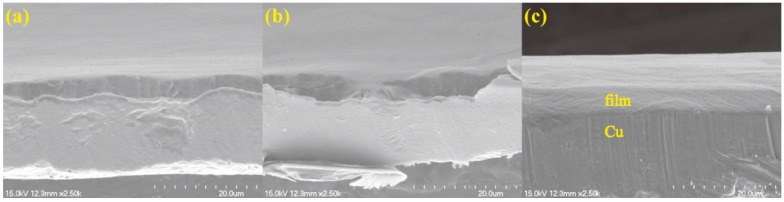
SEM cross-sectional views of carbon films on copper foils with different growth time at ethanol flow rate of 2 mL/min and height of 14.0 mm: (**a**) 30 s (E3-6); (**b**) 2 min (E3-7); (**c**) 5 min (E3-4).

According to the optical microscope images in Figure 4, when the ethanol flow rate was 2 mL/min and the copper foil was inserted into the top of the flame, the growth times of 30 s, 2 min and 5 min were more suitable for the synthesis of carbon films. Figure 10 showed SEM cross-sectional views of the synthetic carbon films with the growth times of 30 s, 2 min and 5 min. The thickness values of these carbon films were 4.13 ± 0.16 μm, 4.52 ± 0.36 μm and 4.68 ± 0.44 μm, respectively. The thickness of the carbon films slightly increased with the increase in growth time.

**Figure 11:**
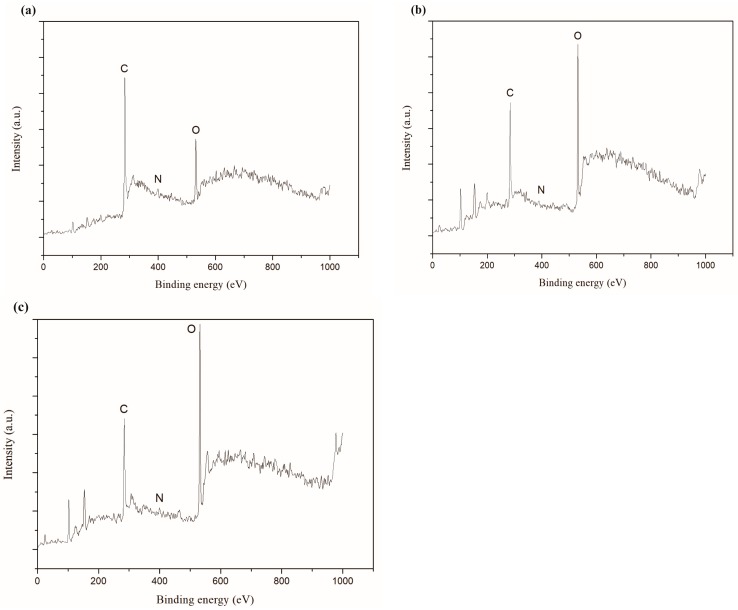
XPS full spectra of carbon films on copper foils with different growth time at ethanol flow rate of 2 mL/min and height of 14.0 mm: (**a**) 30 s (E3-6); (**b**) 2 min (E3-7); (**c**) 5 min (E3-4).

XPS full spectra of the synthetic carbon films with the growth times of 30 s, 2 min and 5 min were exhibited in Figure 11. The results showed that the carbon films mainly contain carbon, nitrogen and oxygen, and the specific corresponding data of relative element molar ratio were listed in Table 3. XPS could not detect hydrogen. Physical adsorption of oxygen and nitrogen during the carbon films exposed to air was the probable reason for the presences of oxygen and nitrogen. In addition, the other reason for the higher oxygen content on the carbon films surfaces might be the formation of C-O functional group during etching process of the copper foils [41]. Small peaks of silicon could be seen in Figure 11b,c because the carbon films on Si/SiO_2_ substrate might have some cracks.

In summary, based on all analysis above, the quality of the synthetic amorphous carbon film was better when the ethanol flow rate reached 2 mL/min, the copper foil was located on the top of the flame and the growth time was 2 min. The resulting carbon film was uniform and intact with higher disorder degree and lower roughness.

## 4. Conclusions

In this article, the flame synthesis of amorphous carbon film on copper foil in a short time with ethanol as fuel in open environments was investigated. The effects of fuel flow rate, the height of copper foil and growth time were considered in detail. The results obtained were summarized as follows:

(1) In open-atmosphere environments, utilizing inverse diffusion flame, with ethanol as fuel and copper foil as synthesis substrate, amorphous carbon film emerged at only 10 s. The synthetic carbon film was ultra-thin and had flexibility, visual transparency to a certain level.
(2) When the growth time was constant, the fuel flow rate and the location of copper foil inserted into the flame had large influences on the synthesis. As the fuel flow rate increased and the copper foil moved upwards in the flame, the area of the carbon film increased, and the content of impurities reduced. In these experiments, the growth of the carbon film was better when the ethanol flow rate reached 2 mL/min and the copper foil was inserted into the top of the flame.
(3) When the fuel flow rate and the copper foil location were optimal, the local growth of amorphous carbon film had occurred in such a short time of 10 s. The content of sp^2^ bonds was higher, the structure of entire carbon film was loose, and the surface was not smooth. When the growth time was 30 s, the content of sp^3^ bonds in the carbon film was maximum, the disorder degree increased, and RMS roughness was smallest. With the increase of the growth time, the carbon film gradually covered the entire surface of the copper foil, the growth of the carbon film almost stopped. The degree of order increased due to the high temperature in the flame for a longer time, and the degree of graphitization rose. When the growth time increased to 10 min, there were some obvious impurities on the carbon film surface, and the roughness of the carbon film was largest.

## Figures and Tables

**Table 1:** Experimental conditions.

Experimental Conditions	Ethanol Flow Rate (mL/min)	Gas Flow Rate (L/min)			Height (mm)	Growth Time (min)
		Air	Carrier N_2_	Protection N_2_		
E1-1	1.0	0.35	1.54	18	3.0	5
E1-2					6.0	
E1-3					10.0	
E1-4					12.0	
E2-1	1.5	0.35	2.31	18	3.5	5
E2-2					6.5	
E2-3					11.0	
E2-4					13.0	
E3-1	2.0	0.35	3.08	18	4.0	5
E3-2					7.0	
E3-3					12.0	
E3-4					14.0	
E3-5					14.0	1/6
E3-6					14.0	1/2
E3-7					14.0	2
E3-8					14.0	10

**Table 2:** The corresponding data of Raman spectra.

Growth Time	Peak Position (cm^−1^)		Ratio of Peak Areas	FWHM (cm^−1^)
	G	D	I_D_/I_G_	G
10 s	1601	1361	2.54	88.6
30 s	1593	1356	1.98	109.6
2 min	1598	1351	2.16	98.9
5 min	1603	1351	4.56	74.4
10 min	1606	1359	6.00	68.6

**Table 3:** The corresponding data of XPS spectra.

Growth Time	C (at.%)	N (at.%)	O (at.%)
30 s	82.88	1.30	15.83
2 min	61.08	1.18	37.74
5 min	54.25	1.37	44.37
